# Generalized Polarimetric Dehazing Method Based on Low-Pass Filtering in Frequency Domain

**DOI:** 10.3390/s20061729

**Published:** 2020-03-20

**Authors:** Jian Liang, Haijuan Ju, Liyong Ren, Liming Yang, Rongguang Liang

**Affiliations:** 1 State Key Laboratory of Transient Optics and Photonics, Xi’an Institute of Optics and Precision Mechanics, Chinese Academy of Sciences, Xi’an 710119, China; juhaijuan@opt.ac.cn (H.J.); yjl890215@sina.com (L.Y.); 2 James C. Wyant College of Optical Sciences, University of Arizona, 1630 E University Blvd, Tucson, AZ 85721, USA; rliang@optics.arizona.edu; 3 School of Physics and Information Technology, Shaanxi Normal University, Xi’an 710119, China; renliy@snnu.edu.cn

**Keywords:** polarimetric imaging, dehazing, frequency domain

## Abstract

Polarimetric dehazing methods can significantly enhance the quality of hazy images. However, current methods are not robust enough under different imaging conditions. In this paper, we propose a generalized polarimetric dehazing method based on low-pass filtering in the frequency domain. This method can accurately estimate the polarized state of the scattering light automatically without adjusting bias parameters. Experimental results show the effectiveness and robustness of our proposed method in different hazy weather and scattering underwater environments with different densities. Furthermore, computational efficiency is enhanced more than 70% compared to the polarimetric dehazing method we proposed previously.

## 1. Introduction

Image dehazing is very critical to obtain clear images in hazy weather [1] and scattering underwater environments [2]. Many techniques have been proposed to enhance the quality of hazy images through image processing [3], infrared-visible image fusion [4] and polarimetric imaging [5,6,7,8]. These techniques have also been combined together, such as polarimetric imaging with image processing [9,10] and polarimetric imaging with image fusion [11], to further improve the performance. These techniques can effectively enhance the contrast of hazy images captured in scattering media, and even increase the imaging distance [2,12]. Among these dehazing techniques, image-processing techniques typically need only one hazy image to do the dehazing process [13,14,15]. Some of them are based on contrast enhancement, which is computationally efficient but less robust. Others are mainly based on a physical degradation model, which is time consuming because it is difficult to estimate appropriate radiance attenuation parameters in one hazy image. By contrast, polarimetric dehazing techniques use several polarized images to estimate the parameters of the scattering light, which have advantages of better image quality and higher efficiency [16,17].

Polarimetric dehazing techniques use intensity information of polarized hazy images to estimate the polarized property of the scattering light and then estimate the intensity of final dehazed images by removing the scattering light from hazy images. Therefore, the accuracy of retrieving the polarized property of the scattering light directly determines the quality of the dehazed images. Normally, in a passive imaging environment, the degree of polarization (DoP) of the scattering light is very small, and easily affected by the quantum noise of the camera sensor. As a result, the DoP of the scattering light usually cannot be estimated accurately, and the quality of the dehazed images is unstable accordingly. To overcome this problem, the DoP of the scattering light is usually tuned by a bias parameter to improve the quality of the dehazed image [5,12,18]. However, this bias parameter is affected by the camera itself and is related to different scattering media, so it is not a fixed value and must be tuned manually in the dehazing process. An image evaluation function can be applied to monitor the quality of dehazed images automatically when the bias parameter is varied [19], but it is still very inconvenient and time consuming.

To overcome this problem, we propose a generalized polarimetric dehazing method by estimating the polarized property of the scattering light after low-pass filtering in the frequency domain. Low-pass filtering in the frequency domain can eliminate most of the quantum noise, which has been widely used in polarimetric imaging techniques [20,21,22,23], and possibly fixes the bias parameters used in the image degradation model. It has been verified as being very effective at roughly separating the information of the scene and the haze in the frequency domain, however, frequency-domain-based dehazing methods are usually complicated. For example, in [6], the researchers use wavelet transform in the frequency domain to enhance the quality of dehazed images. In this paper, we only apply the low-frequency domain to calculate the DoP and no more complicated process is needed. In addition, the computational efficiency is further improved by optimizing the estimation of the angle of polarization (AoP) of the scattering light. Experimental results show that high-quality dehazed images can be obtained in very different hazy weather cases with different camera sensors, as well as in the scattering underwater environment using exactly the same polarimetric dehazing algorithm.

## 2. Theory

### 2.1. Image Degradation Model

In a hazy environment, incident light impinging on the sensor can be divided into two parts [15]. One part of the light is the direct light, which comes from the target object through the haze; the other part is the airlight (the scattering light), which is scattered directly by the haze particles. Thus, the intensity of the light captured by the camera *I* can be expressed by:

(1) I=D+A,

where *D* represents the direct light, and *A* represents the airlight. *D* is the degraded version of the object light (represented by *L*) at a distance of *z*, where the object light is what we need to retrieve. *A* is increased with the distance, and *A* from the infinite distance is usually defined as *A*_∞_. Thus, Equation (1) can be rewritten as:

(2) $$I=L\cdot t\left(z\right)+A_{\infty }\cdot \left[1-t\left(z\right)\right],$$

where *t*(*z*) represents the attenuation process along with the distance, which is usually called as the transmittance of atmosphere [5]. From Equations (1) and (2), one can substitute *t*(*z*) as *D*/*L*, and then *L* can be derived as [5]:

(3) $$L=\frac{I-A}{1-A/A_{\infty }}.$$

When *A* and *A*_∞_ are obtained, the clear images without the influence of the haze can be finally reconstructed.

### 2.2. Fundamental Algorithm of Polarimetric Dehazing

Normally, the scattering process by haze particles satisfies the Mie scattering theory, which means the airlight *A* is always partially polarized light although its DoP may be very small. Therefore, one can estimate *A* accurately using the polarimetric imaging method theoretically. The general process for estimating *A* is explained as follows.

Four polarized images are first captured by the polarimetric imaging system. They can be represented as *I*(0; *x*, *y*), *I*(45; *x*, *y*), *I*(90; *x*, *y*) and *I*(135; *x*, *y*), where the number represents the polarized orientation of the linear polarizer and (*x*, *y*) represents the coordinate of the pixels. Then, the Stokes vector can be obtained as:

(4) $$\begin{array}{c} S_{0}\left(x,y\right)=\left[I\left(0;x,y\right)+I\left(45;x,y\right)+I\left(90;x,y\right)+I\left(135;x,y\right)\right]/2\\ S_{1}\left(x,y\right)=I\left(0;x,y\right)-I\left(90;x,y\right)\\ S_{2}\left(x,y\right)=I\left(45;x,y\right)-I\left(135;x,y\right) \end{array}.$$

The AoP and the DoP can be expressed, respectively, from Equation (4) as:

(5) $$\theta \left(x,y\right)=\frac{1}{2}\arctan\left[\frac{S_{2}\left(x,y\right)}{S_{1}\left(x,y\right)}\right],$$

(6) $$p\left(x,y\right)=\frac{\sqrt{S_{1}{\left(x,y\right)}^{2}+S_{2}{\left(x,y\right)}^{2}}}{S_{0}\left(x,y\right)}.$$

By locating the sky area of hazy images, the AoP and DoP of the airlight can be obtained, which can be represented as *θ*_A_ and *p*_A_. Several methods have been proposed to identify the sky area automatically [19,24]. *A*_∞_ can also be estimated from the highest intensity of the sky area. We also proposed a method to estimate these parameters if there is no sky area in hazy images, in our previous work [25]. Typically, we can assume that *A*_∞_ is a constant for a hazy image, but *A* is a matrix because different pixels of the camera sensor capture the light from different distances. Assuming that the direct light is unpolarized due to the depolarized effect during the scattering process, one can use *θ*_A_ and *p*_A_ to estimate *A* for every pixel of the hazy image [5]. In [25], we proposed an effective way to estimate *A*, expressed as:

(7) $$A\left(x,y\right)=\frac{I\left(0;x,y\right)-S_{0}\cdot \left[1-p\left(x,y\right)\right]/2}{p_{A}\cdot \cos^{2}\theta_{A}}=\frac{I\left(90;x,y\right)-S_{0}\cdot \left[1-p\left(x,y\right)\right]/2}{p_{A}\cdot \sin^{2}\theta_{A}}.$$

According to *θ*_A_, one can choose one term in Equation (7) to estimate *A* without excessively amplifying the noise due to the extremely small denominator.

With *A* and *A*_∞_ obtained by the polarimetric imaging technique, the dehazed image can be reconstructed from Equation (3).

### 2.3. Polarimetric Dehazing Method Based on Low-Pass Filtering in Frequency Domain

From the Mie scattering theory, it is known that the polarized part of the airlight is very small, which means that *S*_1_ and *S*_2_ calculated by Equation (4) are small and may be dominated by the quantum noise of the camera. Normally, the DoP of the airlight is multiplied by a bias parameter to reduce this effect. Besides, *A*_∞_ is defined as *A* from the infinite distance, so *A*_∞_ should be the largest value in *A*. However, due to the quantum noise, *A* calculated by Equation (7) may have larger values than the estimated *A*_∞_. These effects may lead to invalid pixel values in Equation (3). Therefore, to obtain a better dehazed image, another bias parameter is usually applied on *A*_∞_. The bias parameters can reduce the effect of the noise and help to obtain more well dehazed images, but they cause the polarimetric dehazing method to be less robust and effective because the bias parameters must be specified for different hazy environments or different polarimetric imaging systems.

To overcome this problem, we propose a generalized polarimetric dehazing method, which consists of three steps. Firstly, we estimate the AoP of the airlight. In hazy images, because the direct light is the forward-scattering light but the airlight is the integration of the scattering light from all directions, the direct light is depolarized by haze particles and can be treated as non-polarized light, while the airlight is partially polarized due to the multi-scattering effect. Moreover, it can be seen from Equations (4) and (5) that the AoP is only determined by the polarized part of the light. This implies that the direct light has no influence on the AoP estimation. Therefore, the only issue we need to be concerned about is the quantum noise randomly distributed in *S*_1_ and *S*_2_ in a practical process. To avoid the effect of the noise and accurately estimate the AoP of the airlight, we average the values of all pixels to suppress the noise in *S*_1_ and *S*_2_, respectively, to calculate the AoP, expressed as:

(8) $$\theta_{A}=\frac{1}{2}\arctan\left[\frac{\sum_{x=1,y=1}^{m,n}S_{2}\left(x,y\right)}{\sum_{x=1,y=1}^{m,n}S_{1}\left(x,y\right)}\right],$$

where *m* and *n* are the total pixel number in *x* and *y* directions, respectively. This step will help to make the whole estimation process of the AoP faster and more effective, compared with custom methods.

Then, we estimate the DoP of the airlight. From Equation (6), it can be seen that the DoP of the airlight can only be calculated from the sky area, where there is no direct light component in *S*_0_. However, the calculated DoP of the sky area is also severely influenced by the noise [12]. In the frequency domain of hazy images, the airlight is mostly located in the low-frequency region, while the direct light and noise are usually located in the high-frequency region. Therefore, we can apply a low-pass filtering process in the frequency domain to roughly separate the airlight components from the hazy image to further estimate the DoP of the airlight. This process has the advantages that the sky region is not required, and the noise is fully suppressed. The four polarized hazy images are filtered by a low-pass filter in the patch of 5 × 5 in the frequency domain, expressed as:

(9) $$\begin{array}{c} F\left(0;x,y\right)={ℱ}^{-1}\left\{ℱ\left\{I(0;x,y)\right\}\times L\left(5,5\right)\right\}\\ F\left(45;x,y\right)={ℱ}^{-1}\left\{ℱ\left\{I(45;x,y)\right\}\times L\left(5,5\right)\right\}\\ F\left(90;x,y\right)={ℱ}^{-1}\left\{ℱ\left\{I(90;x,y)\right\}\times L\left(5,5\right)\right\}\\ F\left(135;x,y\right)={ℱ}^{-1}\left\{ℱ\left\{I(135;x,y)\right\}\times L\left(5,5\right)\right\} \end{array},$$

where F{} and F^-1^{} represent the Fourier transform and the inverse Fourier transform, respectively. *L* represents the low-pass filter, (5, 5) represents the patch is 5 × 5. It should be pointed out that 5 × 5 is a recommended region after the experiments, since a larger region may contain more information of the scene, while a smaller region may lose all the information of the image. Similar to Equations (4)–(6), the expressions of the Stokes vector, the AoP and the DoP can be written as:

(10) $$\begin{array}{c} S_{0\_F}\left(x,y\right)=\left[F\left(0;x,y\right)+F\left(45;x,y\right)+F\left(90;x,y\right)+F\left(135;x,y\right)\right]/2\\ S_{1\_F}\left(x,y\right)=F\left(0;x,y\right)-F\left(90;x,y\right)\\ S_{2\_F}\left(x,y\right)=F\left(45;x,y\right)-F\left(135;x,y\right) \end{array},$$

(11) $$\theta_{F}\left(x,y\right)=\frac{1}{2}\arctan\left(\frac{S_{2\_F}\left(x,y\right)}{S_{1\_F}\left(x,y\right)}\right),$$

(12) $$p_{F}\left(x,y\right)=\frac{\sqrt{S_{1\_F}{\left(x,y\right)}^{2}+S_{2\_F}{\left(x,y\right)}^{2}}}{S_{0\_F}\left(x,y\right)}.$$

The images after low-pass filtering are dominated by the airlight, so the whole images can be roughly regarded as the sky region. To accurately estimate the DoP of the airlight, we use *θ*_A_ calculated by Equation (8) to determine the effective pixels in the map *θ*_F_. The largest value of the DoP among all the effective pixels in the map *p*_F_ is the estimated DoP of the airlight *p*_A_. Once the AoP and the DoP are determined, we can estimate the airlight *A* using Equation (7). Here, we only use a low-pass filter to roughly estimated the airlight map, and no more complicated process is needed, which is effective in accurately estimating the *p*_A_ without a bias parameter, and besides, remains high computational efficiency.

Finally, we estimate *A*_∞_. Normally, *A*_∞_ is estimated from the sky area, however, this estimated value may be smaller than that of some pixels in *A*, which will lead to an invalid dehazing result, according to Equation (3). Therefore, in a practical process, only using the sky area to estimate *A*_∞_ seems to be less robust. In fact, *A*_∞_ can be regarded as one specific value of the airlight, so if *A* is accurately estimated, the largest value of the *A* can be considered as *A*_∞_. It can be further analyzed from Equation (3) that *A*_∞_ should be a little larger than the largest value of *A*, to avoid the denominator becoming 0. In our case, the *A*_∞_ is expressed as:

(13) $$A_{\infty }=1.02\times \max\left[A\left(x,y\right)\right].$$

The coefficient 1.02 is used to ensure *A*_∞_ slightly larger than the largest value of *A*. Note that, the dehazed result is mainly determined by *A*, not *A*_∞_. So, this coefficient is not critical as long as it is slightly larger than 1, which we will discuss later. When *A* and *A*_∞_ are obtained, the dehazed result can be calculated using Equation (3).

**Figure 1:**
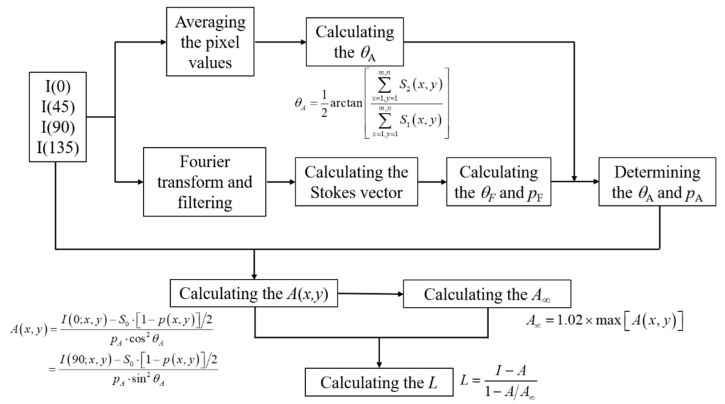
The workflow of the proposed polarirmetric dehazing method.

Since we estimate AoP and DoP of the airlight without the influence of the noise, their accuracies are maintained in different scattering media, and it is not necessary to manually tune bias parameters during the dehazing process. Therefore, the proposed polarimetric dehazing method is robust and can adapt in different scattering media or different polarimetric imaging systems. The workflow of the proposed method is shown as Figure 1. It can be seen from Figure 1 that the whole dehazing process is quite simple compared with other frequency-domain-based dehazing methods, while no bias parameters need to be tuned manually to obtain good dehazed results. Experimental results show that good dehazed results can be obtained in different scattering media, with high computational efficiency. From Figure 1, it can also be seen that we only use the frequency domain to accurately estimate the AoP and DoP of the airlight, while the main dehazing process is carried on with original hazy images, therefore, the detailed information of the image will not be blurred due to the low-pass filtering in the frequency domain.

## 3. Experiments and Discussion

**Figure 2:**
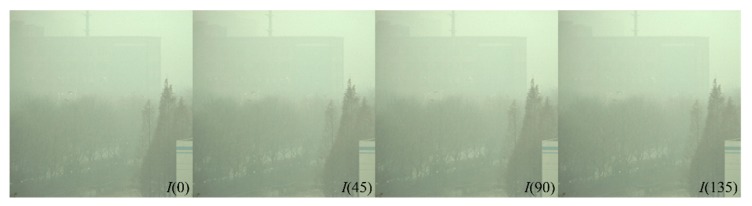
Original captured polarized images in very severe hazy weather.

**Figure 3:**
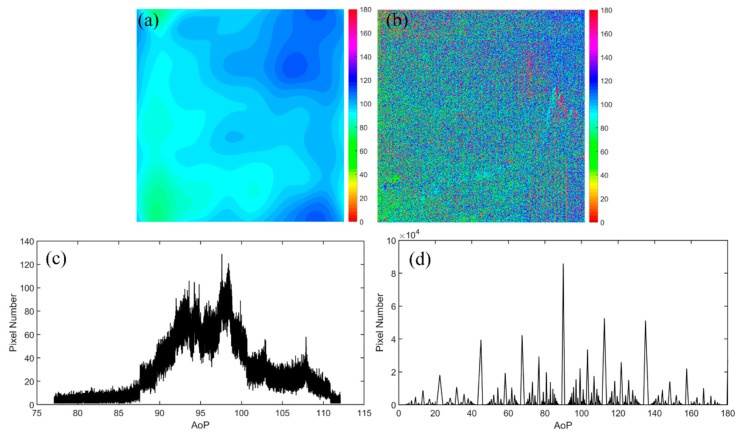
The AoP distribution of the hazy images: (**a**) after low-pass filtering and (**b**) before low-pass filtering. Statistical distribution: (**c**) after and (**d**) before low-pass filtering.

We first use a group of experiments captured in very severe hazy weather to demonstrate the effectiveness of the proposed method. The original captured polarized images are shown in Figure 2. Firstly, we calculate the AoP of the airlight using Equation (8), *θ*_A_ is 97.14°. Then we calculate the distribution of the AoP after filtering Figure 2 using Equation (9) and the result is shown in Figure 3a. For comparison, we also calculate the distribution of the AoP directly using Figure 2, as shown in Figure 3b. In Figure 3a, the distribution of the AoP is smooth, meaning that the quantum noise is suppressed, However the distribution of the AoP in Figure 3b is random. Furthermore, the statistical distributions are also given, as shown in Figure 3c,d after and before the low-pass filtering, respectively. It is clearly seen that the range of the AoP in Figure 3c is 77°–112°, while due to the noise, the range in Figure 3d is 0°–180°. The peak value in Figure 3c is 97.61°, which is very close to our calculated *θ*_A_. This indicates that our assumption, i.e., images after low-pass filtering are dominated by the airlight, is reasonable.

**Figure 4:**
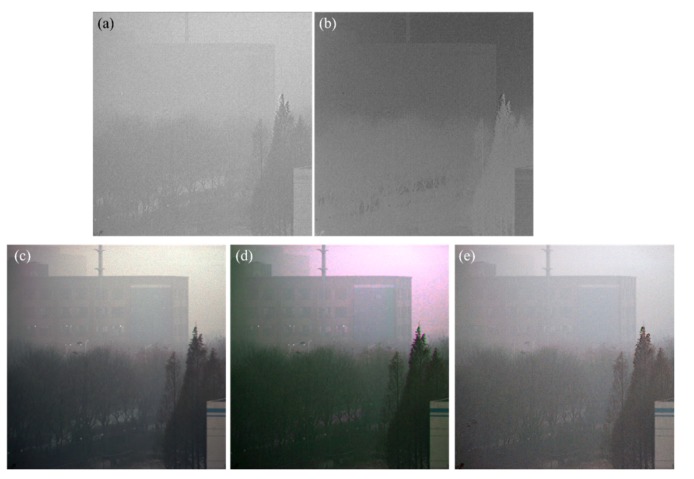
(**a**) The map of *A*; (**b**) the transmittance of atmosphere; (**c**) the dehazed image using our proposed polarimetric dehazing method; (**d**) the dehazed image using the dehazing method proposed in [12]; (**e**) the dehazed image using the dehazing method proposed by Y. Schechner [24].

Then we use *θ*_A_ to determine the effective pixels of Figure 3a and use the maximum value of the DoP in all the effective pixels as the *p*_A_. Using Equation (7), we can estimate the map of *A*, as shown in Figure 4a. *A*_∞_ is then estimated using Equation (13). We show the transmittance of atmosphere in Figure 4b to illustrate that the estimated *A*_∞_ is appropriate. From Figure 4b, it can be seen that the nearer scene (trees) has a brighter value, while the farther scene (building) has a relatively darker value.

Finally, we can reconstruct the dehazed image with the estimated *A* and *A*_∞_, as shown in Figure 4c. It can be seen that the contrast of the image is enhanced a lot. The visibility of the image is also enhanced, especially for the long-distance building in the top-left corner, which is hardly seen in the original hazy image. This experimental result illustrates the effectiveness of the proposed polarimetric dehazing method. As a comparison, Figure 4d is the reconstructed image using our previously proposed polarimetric dehazing method [12]. Both methods can reconstruct high-quality dehazed images, but it is necessary to tune the bias parameters manually in our previous method. To demonstrate the good quality of the dehazed image using the proposed method, we also compared the dehazing result to that using Y. Y. Schechner’s proposed method [24], as shown in Figure 4e. It can be seen that the quality of Figure 4c,d is better than that of Figure 4e. Note that, the main advantage of the dehazing method proposed in [24] is that it can automatically identify the sky region, however, in their method, the bias parameters still need to be adjusted manually in different hazy environments.

Here, we apply the image contrast to evaluate the contrast enhancement of the dehazed image objectively [26], which can be written as:

(14) $$C\left(I\right)=\frac{\sqrt{\frac{1}{N}\sum_{x,y}{\left[I\left(x,y\right)-\bar{I}\right]}^{2}}}{\bar{I}},$$

where *N* is the number of pixels in the image, $\bar{I}$ is the mean intensity of the image. The contrast of the hazy image is 0.1673, while the contrast of Figure 4c,d are 0.5375 and 0.5823, respectively. It can be seen that the improvement of the dehazed result using the proposed method is approximately the same as that using the previous proposed method with appropriate bias parameters. Both of the dehazed results have a very good performance compared with the hazy image. The contrast of Figure 4e is 0.3748, which is much less than the proposed method. Moreover, the dehazing capability of the previous proposed method with other dehazing methods has been fully compared in [27], so in the following, we mainly focus on the effectiveness and robustness of the proposed method with the previous method in the discussion.

**Figure 5:**
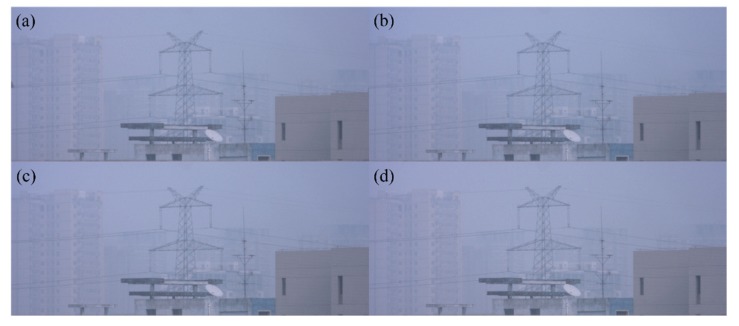
Original captured images in different hazy weather. (**a**–**d**) represent the raw hazy images with the polarized orientation of 0°, 45°, 90° and 135°, respectively.

**Figure 6:**
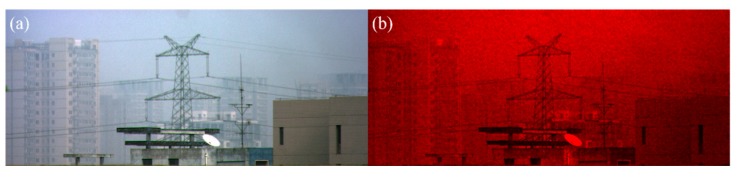
The dehazed images using (**a**) the current proposed method; (**b**) our previously proposed method with the fixed bias parameters.

Although both the dehazing methods can obtain good dehazed results, the proposed method does not need to manually tune any bias parameter and thus it is robust. To demonstrate its robustness, the proposed polarimetric dehazing method is used to process the polarized images (Figure 5) captured in a very different hazy weather. Figure 6a,b are the dehazed results using the current proposed method and the previously proposed method with the same bias parameter as that used in Figure 4. We can see that the contrast of Figure 6a is enhanced a lot compared with Figure 5. However, since the bias parameter is not fine-tuned to this group of hazy images, the dehazed result (Figure 6b) of the previously proposed method is much worse than Figure 6b. Note that we can still obtain a better dehazed image using the previously proposed method with the appropriate bias parameter as discussed in [12]. Besides, in Figure 6b, the red background implies that there may be huge errors when calculating the other two channels (green and blue channels). It usually appears when the bias parameters are not suitable for the hazy image.

**Figure 7:**
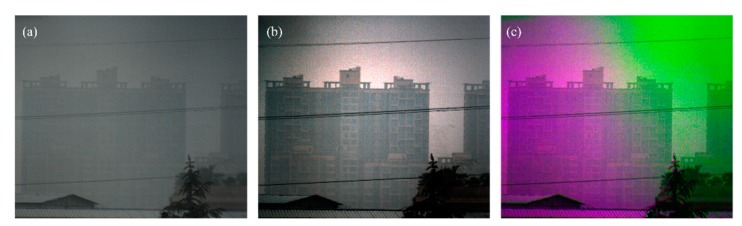
The dehazing experimental results. (**a**) Original intensity image; (**b)** dehazed image using the current proposed method; and (**c**) dehazed image using our previously proposed method with the fixed bias parameters.

Figure 7a is one of the original polarized images captured with our designed division-of-aperture type polarimetric imaging camera [16]. The transmittance of the imaging lens is not calibrated, and the surrounding pixels of the sensor may be a little darker than the center. This may affect the accuracy of the calculated polarized property. We use this experiment to further show the robustness of the proposed method. Due to the reason that the response of the sensor is different, it is hard to estimate the accurate DoP and AoP based on the raw images, even with tuned bias parameters. Figure 7b,c are the dehazed results using the current proposed method and the previously proposed method with fixed bias parameters, respectively. It can be seen that the quality of Figure 7b is much better than that of Figure 7a,c. The top region in Figure 7b is the sky region, where the value of *A* is close to *A*_∞_. However, similar to Figure 6b, Figure 7c shows strange colors in different regions, which implies the bias parameters used in red green blue (RGB) channels may also be different to use of the previous method. As a contrast, we don’t need to set the bias parameter in the current method, but good dehazed result can be obtained.

**Figure 8:**
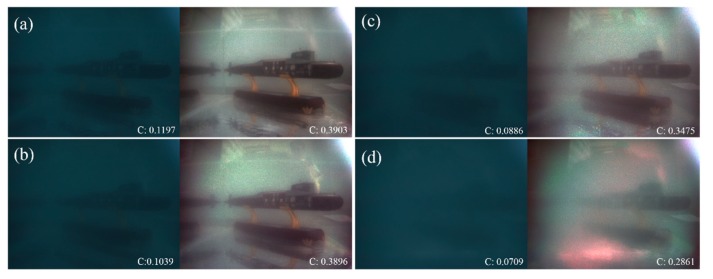
Underwater descattering experimental results. (**a**)–(**d**) are the original intensity images (left one of each group) and dehazed images (right one of each group) from light scattering to dense scattering conditions.

In addition to processing the images under different hazy weather, we also evaluate the performance of the proposed polarimetric dehazing method in processing the images under the scattering underwater environment. We use the submarine model as the target and simulate different scattering conditions with magnesium oxide (MgO). In this experiment, another pixelated underwater polarization camera is used to capture images. Figure 8a–d show the original intensity images (left) and the dehazed images (right) under different scattering conditions. It can be seen that the image quality can be improved significantly at all scattering conditions. The contrasts of the images are also shown in Figure 8. It can be seen that all the contrast of the dehazed images are enhanced a lot, over 3 times compared with that of the hazy images.

**Figure 9:**
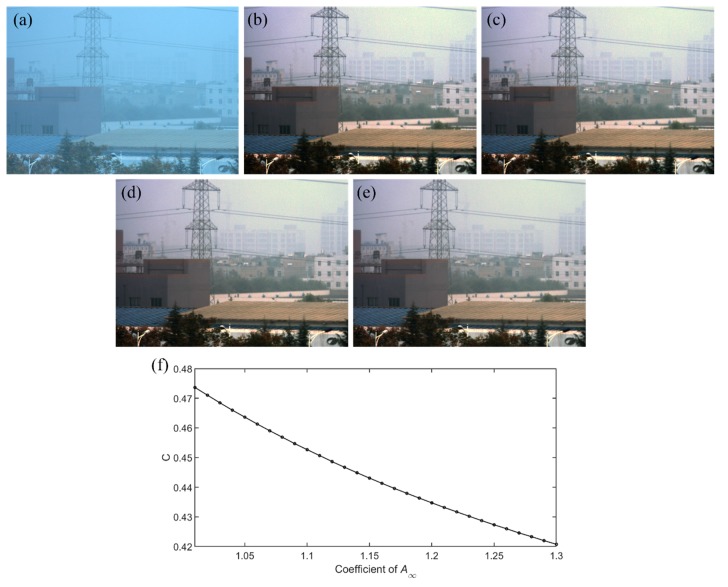
Experimental results with different coefficients of *A*_∞_. (**a**) Original intensity image; (**b**)–(**e**) dehazed images with the coefficient of 1.01, 1.05, 1.2 and 1.3, respectively; and (**f**) the relationship between the contrast (C) and the coefficient of *A*_∞_.

In the proposed polarimetric dehazing method, we assume *A*_∞_ to be a little larger than the maximum value of *A*. We use an image set with 20 groups of hazy images captured in different scattering media to validate this assumption, and it is found that the results are the same. Therefore, we choose one group of experiments captured in the different hazy conditions compared with the examples shown above to demonstrate the validity of this assumption, while further showing the effectiveness of the proposed method. Figure 9 shows the experimental results with different coefficients of *A*_∞_. Figure 9a is the original hazy image with the contrast of 0.1763. The dehazed images, as shown in Figure 9b–e, are those using our proposed dehazing method with different coefficients of 1.01, 1.05, 1.2 and 1.3, respectively. It can be seen from Figure 9f that the contrast of the dehazed image decreases slowly as the coefficient increases, which implies that the coefficient has a very large tolerance when choosing. For the best performance, the coefficient should be slightly larger than 1, and in our experiments, we fix this coefficient as 1.02.

The proposed polarimetric dehazing method can effectively enhance the image quality under different imaging conditions. With optimized algorithms to estimate the AoP and the DoP of the airlight, the computational efficiency is also significantly enhanced. Table 1 summarizes the process time measured in Matlab R2017b on a personal computer with an Inter(R) Core(TM) i7-4910MQ CPU @2.90 GHz processor and 16 G RAM. We compare the current proposed method to the method we originally proposed in [12]. It can be seen that the computational efficiency is improved by more than 70%.

## 4. Conclusions

Most polarimetric dehazing methods use bias parameters to address the issue related to the quantum noise of the sensor in order to obtain high-quality dehazed images, and these bias parameters should be tuned in different scattering environments or in RGB channels, which make the polarimetric dehazing methods less robust. In this paper, we propose an optimized algorithm to accurately estimate the AoP and the DoP of the airlight without the bias parameters. We have also improved the computational efficiency more than 70% compared to the previously proposed method. Experimental results have demonstrated the effectiveness and robustness of our proposed method under different imaging condition and using different imaging systems.

## Figures and Tables

**Table 1:** Comparison of the process time for two polarimetric dehazing methods.

Scene	Spatial Resolution	Execution Time (s)		Enhancement (%)
		Our Proposed Method	Method Proposed in [12]	
Scene 1 (Figure 2)	998 × 1023	1.53	2.77	81.04
Scene 2 (Figure 5)	1352 × 505	0.89	1.69	89.89
Scene 3 (Figure 7)	898 × 786	1.07	1.96	83.18
Scene 4 (Figure 8)	1360 × 1024	1.93	3.66	89.64
Scene 5 (Figure 9)	997 × 718	1.03	1.77	71.84
